# Catastrophic expenditure on medicines in Brazil

**DOI:** 10.1590/S1518-8787.2016050006172

**Published:** 2016-12-01

**Authors:** Vera Lucia Luiza, Noemia Urruth Leão Tavares, Maria Auxiliadora Oliveira, Paulo Sergio Dourado Arrais, Luiz Roberto Ramos, Tatiane da Silva Dal Pizzol, Sotero Serrate Mengue, Mareni Rocha Farias, Andréa Dâmaso Bertoldi

**Affiliations:** I Departamento de Política de Medicamentos e Assistência Farmacêutica. Escola Nacional de Saúde Pública Sérgio Arouca. Fundação Oswaldo Cruz. Rio de Janeiro, RJ, Brasil; II Departamento de Farmácia. Faculdade de Ciências da Saúde. Universidade de Brasília. Brasília, DF, Brasil; III Departamento de Farmácia. Faculdade de Farmácia, Odontologia e Enfermagem. Universidade Federal do Ceará. Fortaleza, CE, Brasil; IV Departamento de Medicina Preventiva. Escola Paulista de Medicina. Universidade Federal de São Paulo. São Paulo, SP, Brasil; V Departamento de Produção e Controle de Medicamentos. Faculdade de Farmácia. Universidade Federal do Rio Grande do Sul. Porto Alegre, RS, Brasil; VI Programa de Pós-Graduação em Epidemiologia. Faculdade de Medicina. Universidade Federal do Rio Grande do Sul. Porto Alegre, RS, Brasil; VII Departamento de Ciências Farmacêuticas. Centro de Ciências da Saúde. Universidade Federal de Santa Catarina. Florianópolis, SC, Brasil; VIII Departamento de Medicina Social. Faculdade de Medicina. Universidade Federal de Pelotas. Pelotas, RS, Brasil

**Keywords:** Drug Price, Health Expenditures, Family Characteristics, Socioeconomic Factors, Health Surveys

## Abstract

**OBJECTIVE:**

To describe the magnitude of the expenditure on medicines in Brazil according to region, household size and composition in terms of residents in a situation of dependency.

**METHODS:**

Population-based data from the national household survey were used, with probabilistic sample, applied between September 2013 and February 2014 in urban households. The expenditure on medicines was the main outcome of interest. The prevalence and confidence intervals (95%CI) of the outcomes were stratified according to socioeconomic classification and calculated according to the region, the number of residents dependent on income, the presence of children under five years and residents in a situation of dependency by age.

**RESULTS:**

In about one of every 17 households (5.3%) catastrophic health expenditure was reported and, in 3.2%, the medicines were reported as one of the items responsible for this situation. The presence of three or more residents (3.6%) and resident in a situation of dependency (3.6%) were the ones that most reported expenditure on medicines. Southeast was the region with the lowest prevalence of expenditure on medicines. The prevalence of households with catastrophic health expenditure and on medicines in relation to the total of households showed a regressive tendency for economic classes.

**CONCLUSIONS:**

Catastrophic health expenditure was present in 5.3%, and catastrophic expenditure on medicines in 3.2% of the households. Multi-person households, presence of residents in a situation of economic dependency and belonging to the class D or E had the highest proportion of catastrophic expenditure on medicines. Although the problem is important, permeated by aspects of iniquity, Brazilian policies seem to be protecting families from catastrophic expenditure on health and on medicine.

## RESUMO

**OBJETIVO:**

Descrever a magnitude do gasto catastrófico em medicamentos no Brasil segundo região, tamanho das famílias e composição familiar em termos de moradores em situação de dependência.

**MÉTODOS:**

Utilizados dados de inquérito domiciliar nacional, de base populacional, com amostra probabilística, aplicado entre setembro de 2013 e fevereiro de 2014 em domicílios urbanos. O gasto catastrófico em medicamentos foi o principal desfecho de interesse. As prevalências e intervalos de confiança de 95% (IC95%) desses desfechos foram estratificados segundo classificação socioeconômica e calculadas de acordo com a região, o número de moradores dependentes da renda, a presença de crianças menores de cinco anos e de moradores em situação de dependência, por idade.

**RESULTADOS:**

Em cerca de um de cada 17 domicílios (5,3%) foi relatado gasto catastrófico em saúde e, em 3,2%, os medicamentos foram reportados como um dos itens responsáveis por esta situação. Presença de três ou mais moradores (3,6%) e morador em situação de dependência jovem (3,6%) foram as situações com maior relato de gasto catastrófico em medicamentos. O Sudeste foi a região com menor prevalência de gasto catastrófico em medicamentos. As prevalências de domicílios com gasto catastrófico em saúde e medicamentos em relação ao total de domicílios apresentaram tendência regressiva para as classes econômicas.

**CONCLUSÕES:**

O gasto catastrófico em saúde esteve presente em 5,3% e o gasto catastrófico em medicamentos, em 3,2% dos domicílios. Domicílios pluripessoais, presença de moradores em situação de dependência econômica e pertencimento à classe D ou E tiveram a maior proporção de gasto catastrófico em medicamentos. Ainda que o problema se mostre importante, permeado por aspectos de iniquidade, as políticas brasileiras parecem estar protegendo as famílias do gasto catastrófico em saúde e em medicamentos.

## INTRODUCTION

The medicines are essential health inputs and determinants in the good outcome of a large number of diseases and harms to health, including the increased survival rate and relief of suffering.

Many countries face serious problems of access both to health services and to medicines^23^. However, both indicators tend to be high in many countries, even among vulnerable groups, although to a greater magnitude in groups of higher income^23^. Wagner et al., reviewing data from 70 countries, showed that between 93.0% and 100% of individuals reported getting health care and from 72.0% to 83.0% all or almost all of them managed to get the medicines they sought for the last 12 months^24^. Study in four Central American countries showed that 79.1% of individuals found access to medicines (sought and obtained)^1^.

The consumption of medicinal products is influenced, inter alia, by market failure^5^. One of them, of particular relevance in this study, is the price inelasticity of demand^21^. Facing the need to use medicines, even if expensive, users are compelled to dispose of property or resort to justice to demand them, in order to ensure continuity of treatment and the mitigation of their suffering.

Thus, an important aspect is to understand the economic impact of this access for families and individuals. Expenditures on health may contribute to the impoverishment of individuals^27^, a fact evidenced also for Brazil^9^. When the ability to pay with their income is exceeded, people count on loans and savings, sell assets, reduce essential expenses, such as food or education^24^, and all these measures have a negative impact on their quality of life.

Catastrophic health expenditure (CHE) has been used to express the excessive financial burden for families. It can be measured in various ways, such as by calculating the proportion of expenditure on health in relation to the total income of the household^4^ ^,^ ^25^, by measuring the ability to pay (affordability)^27^ and by the presence of deleterious impacts on the family budget^14^. We did not find in the literature an operational definition of catastrophic expenditure on medicines (CEM).

Wagner et al. found means of 9.0% and 18.6% of households in upper-middle income countries for CHE, measured, respectively, (a) by the health expenditure of 40.0% or more of household income and (b) by the declaration of people who sold goods or requested loans for medical expenses^24^. The authors observed that between 41.0% and 61.0% of individuals, respectively in households of low- and high-income countries, spent their entire budget for health on medicine^24^. Knaul et al. showed 1.0% to 25.0% of households that reported CHE in study including 12 Latin American countries^13^.

In Brazil, the public provision of medicines occurs by different mechanisms – free provision in public health facilities or by free provision or copayment in the Popular Pharmacy Program. Despite this, medicines are the main responsibles for health expenditure, being around 45.0% of the budget for health of families and charging, especially, the poorest^3^ ^,^ ^12^.

This study aimed to describe, based on data from the national household survey, the magnitude of CEM in Brazil according to regions of the Country, household size, and composition in terms of residents in a situation of dependency.

## METHODS

This article uses data from the household survey conducted as part of the *Pesquisa Nacional sobre Acesso, Utilização e Promoção do Uso Racional de Medicamentos* (PNAUM – National Survey on Access, Use and Promotion of Rational Use of Medicines), which also featured a component implemented in primary health care services. The component of the survey consisted of a cross-sectional population-based study, applied in permanent households of urban areas throughout Brazil. Exactly 20,404 households were included and 41,433 people were interviewed, representing 171 million of residents in urban areas of Brazil. The face-to-face questionnaire, specifically prepared by the team of researchers, was applied by means of an electronic device (tablet), from September 2013 to February 2014. It contained 11 blocks of questions: general information of the respondent; chronic diseases (not infectuous); detail of medicines of continuous use; use of health services; acute diseases; detail of the medicines of eventual use; contraceptives; pharmacy services; behaviors that may affect the use of medicines; package inserts and packages; lifestyle; health plan; and household information. This article uses data from the last block (household information). Details about the method can be found in another article^16^.

The outcomes of interest were CEM (primary outcome) and CHE (secondary outcome). CHE was acknowledged as existent when the question “Did you not buy something to pay expenses with health problems?” received affirmative answers, and CEM, every time the drugs were referred to as one of the items responsible for expenditures on health problem. The choice for such measures happened because the questionnaire did not contemplate questions of financial expenses.

The prevalence and 95% confidence intervals (95%CI) of CHE and CEM – both having as denominator the total of valid households – were stratified according to the *Critério Classificação Econômica Brasil* (CCEB – Brazilian Economic Classification Criterion) developed by the *Associação Brasileira de Empresas de Pesquisa* (ABEP – Brazilian Research Association)^a^ (2013) in “A/B”, “C”, “D/E”. It was estimated according to the regions of the Country; number of residents that depend on the income (1; 2; 3 or more); the presence of children under the age of five; and the presence of residents in situation of dependency by age (under 15 years [young-age dependency ratio] and people aged 65 years or over [old-age dependency ratio]). Whenever the coefficient of variation was greater than 30.0%, caution was recommended in the interpretation of the data. We also presented ways to deal with the CHE according to ABEP’s classification, applying the Pearson’s Chi-square test to assess the statistical significance of differences among groups, whereas a significance level of 5%. All calculations were performed on the weighted database post-stratification to ensure national representation, being made extrapolation for 171 million of inhabitants of urban areas. Data were analyzed with the statistical program SPSS (Inc. Released 2009. Pasw Statistics for Windows, Version 18.0. Chicago: SPSS Inc).

^a^ Associação Brasileira de Empresas de Pesquisa (ABEP). Critério de Classificação Econômica Brasil. Alterações na aplicação do Critério Brasil, válidas a partir de 1/1/2013. São Paulo (SP): Associação Brasileira de Empresas de Pesquisa; 2013 [cited 2016 Aug 1]. Available from: http://www.abep.org/criterio-brasil

The study was approved by the National Committee for Ethics in Research (CONEP – Opinion 398,131, from September 16, 2013) and all the interviews were conducted after the respondents or their legal guardians (in case of minors or people unable to answer their own questionnaire) read and signed the informed consent form.

## RESULTS

Of the total number of 20,404 households visited, 909 households were lost due to the lack of reply to CHE and three others due to insufficient information for the construction of ABEP’s variable socioeconomic classification. Thus, 19,492 households were studied regarding the outcomes of interest. Of these, the majority belonged to the class C (57.3%) and resided in the Southeast region (45.1%). The households had an average of 3.1 residents, the majority (68.2%) with three or more, and the minority (11.6%) with one resident only. About 1/4 of the households had children under the age of five, about half had residents in the young-age dependency ratio (under 15 years) and 20.4% in the old-age dependency ratio (over 65 years). Most (66.7%) households had some resident in situation of dependency when considered both the limits of the age group (Table 1).

**Table 1:** Distribution of households stratified by economy class according to the regions of the Country, number of inhabitants, and the presence of children and residents in situation of dependency by age. PNAUM, Brazil, 2014.

Variable	Proportion of households^a,b^							

	A/B		C		D/E		Total	
			
	%	95%CI	%	95%CI	%	95%CI	%	95%CI
Region								
North	4.8	3.5–6.7	7.8	6.1–9.9	9.2	7.0–12.1	7.4	5.9–9.4
Northeast	10.3	7.7–13.8	24.3	20.0–29.1	44.4	37.5–51.6	25.3	21.0–30.1
Southeast	57.6	50.9–64.0	44.5	38.6–50.4	33.3	26.7–40.7	45.1	39.4–51.0
South	18.2	14.4–22.8	14.8	11.9–18.4	6.7	5.0–9.0	13.9	11.2–17.1
Midwest	9.0	6.8–11.9	8.7	6.8–10.9	6.3	4.7–8.3	8.3	6.5–10.4
Number of residents								
1	6.7	5.2–8.4	11.5	10.2–13.0	17.1	15.2–19.2	11.6	10.5–12.7
2	20.0	17.5–22.7	19.6	18.4–20.8	22.4	20.4–24.5	20.2	19.2–21.3
≥ 3	73.3	70.0–76.5	68.9	66.9–70.9	60.5	57.5–63.4	68.2	66.5–69.9
Situation of dependency by age^c^								
Presence of people under 5 years	10.6	4.9–21.3	28.3	26.5–30.2	30.6	28.0–33.3	27.2	25.7–28.8
Presence of people under 15 years	46.8	43.8–49.9	52.5	50.4–54.5	51.8	48.8–54.7	51.1	49.2–52.9
The presence of people aged 65 years or over	19.9	17.9–22.0	19.9	18.5–21.5	22.3	20.1–24.6	20.4	19.2–21.7
Presence of people under 15 years, 65 years or over	61.8	59.0–64.6	67.7	66.1–69.3	69.1	66.4–71.6	66.7	65.3–68.0

Total	22.3	20.4–24.4	57.3	55.8–58.9	20.3	18.6–22.2	100	-

^a^ The percentages shown were weighted by the sample weights.

^b^ Brazil Economic Classification Criterion developed by the Brazilian Research Association (CCEB 2013/ABEP). Available from: http://www.abep.org

^c^ The percentages correspond to the dichotomous situation, in this case, the absence of a resident in the corresponding condition.

In about one of every 17 households (5.3%) CHE was reported and, in 3.2%, the medicines were reported as one of the items responsible for this situation (value not shown in the Table). The Southeast region showed the lowest prevalence of households with CHE (3.2%) (Table 2).

**Table 2:** Prevalence of households with catastrophic health expenditure stratified by economic class, according to the regions of the Country, number of inhabitants, and presence of children and residents in a situation of dependency by age. PNAUM, Brazil, 2014.

Variable	Prevalence of households where catastrophic health expenditure was declared^a,b^							

	A/B (n = 137)		C (n = 727)		D/E (n = 288)		General (n = 1.152)	
			
	%	95%CI	%	95%CI	%	95%CI	%	95%CI
Region		p < 0.001		p = 0.001		p = 0.072		p < 0.001
North	3.2	1.7–5.8^c^	6.8	5.0–9.2	6.7	4.4–10.2	6.2	4.8–8.1
Northeast	6.9	3.5–13.1^c^	7.8	5.9–10.0	8.3	6.3–10.9	7.9	6.3–9.8
Southeast	1.5	0.8–2.7^c^	3.8	2.6–5.5	4.2	2.2–7.6^c^	3.2	2.4–4.2
South	3.3	2.3–4.6	7.3	5.9–9.0	6.7	4.0–11.0	6.1	5.0–7.3
Midwest	3.5	2.2–5.4	6.7	5.4–8.2	8.8	5.2–14.4	6.2	5.1–7.5
Number of residents		p = 0.016		p < 0.001		p = 0.002		p < 0.001
1	0.8	0.3–1.9^c^	2.6	1.8–3.7	2.8	1.7–4.5	2.4	1.8–3.2
2	1.7	1.0–2.9	5.3	4.2–6.7	6.9	4.7–9.8	4.9	4.0–5.9
≥ 3	3.0	2.2–4.2	6.4	5.3–7.7	7.8	6.0–9.9	5.8	5.0–6.8
Situation of dependency by age								
Under 5 years		p = 0.006		p = 0.749		p = 0.178		p = 0.372
Presence	1.4	0.8–2.3	6.0	4.5–8.0	8.2	5.6–11.8	5.7	4.5–7.1
Absence	3.0	2.1–4.1	5.7	4.9–6.6	6.1	4.7–7.8	5.1	4.5–5.8
Under 15 years		p = 0.064		p = 0.051		p = 0.015		p = 0.001
Presence	3.4	2.1–5.3	6.4	5.2–7.9	8.1	6.2–10.5	6.1	5.2–7.3
Absence	2.0	1.4–2.8	5.0	4.3–5.9	5.2	4.0–6.9	4.3	3.8–5.0
65 years or over		p = 0.121		p = 0.408		p = 0.323		p = 0.628
Presence	3.7	2.5–5.4	5.3	4.4–6.4	5.7	4.1–7.9	5.1	4.3–5.9
Absence	2.4	1.6–3.5	5.9	4.9–7.0	7.0	5.5–8.9	5.3	4.6–6.2
Under 15 years, 65 years or over		p = 0.002		p = 0.325		p = 0.255		p = 0.010
Presence	3.4	2.4–4.8	6.0	5.0–7.2	7.2	5.6–9.1	5.7	4.9–6.6
Absence	1.4	0.9–2.2	5.3	4.4–6.4	5.7	4.0–8.0	3.7	5.2–8.7^c^
General	2.6	1.9–2.2	5.8	5.0–6.7	6.7	5.4–8.3	5.3	4.6–6.0

^a^ The percentages shown were weighted by the sample weights.

^b^ Brazil Economic Classification Criterion developed by the Brazilian Research Association (CCEB 2013/ABEP). Available from: http://www.abep.org

^c^ We recommend caution in the interpretation of the data.

Regarding the number of residents, the higher prevalence of CHE was among households with three or more (5.8%), with significant differences in all economic classes (Table 2).

As for the residents in a situation of economic dependency, we observed a higher prevalence of CHE, statistically significant, with the presence of residents in the young-age dependency ratio (Table 2).

The prevalence of CEM behaved very similar to that of health, with lower prevalence in the Southeast region (1.7%), especially in class A/B (0.9%) (Table 3). When considered the number of residents, the prevalence of two or more was almost twice as high as that of only one resident, pattern that repeated itself for the economic classes C and D/E. The CEM was greater in the presence of young dependency (3.6%) (Table 3).

**Table 3:** Prevalence of households with catastrophic expenditure on medicines stratified by economic class, according to the regions of the Country, number of inhabitants, and presence of children and residents in a situation of dependency by age. PNAUM, Brazil, 2014.

Variable	Prevalence of households where catastrophic expenditure on medicines was declared^a,b^							

	A/B (n = 80)		C (n = 466)		D/E (n = 194)		General (n = 740)	
			
	%	95%CI	%	95%CI	%	95%CI	%	95%CI
Region		p = 0.000		p = 0.000		p = 0.135		p = 0.000
North	2.2	1.0–5.1^c^	4.5	3.2–6.3	5.1	3.1–8.4	4.3	3.2–5.9
Northeast	5.2	2.2–12.1^c^	5.5	4.2–7.1	5.6	4.1–7.6	5.5	4.3–7.0
Southeast	0.9	0.4–2.1^c^	1.8	1.2–2.5	3.0	1.5–5.9^c^	1.7	1.3–2.3
South	2.1	1.4–3.1	3.9	2.9–5.3	5.4	3.0–9.7^c^	3.5	2.7–4.5
Midwest	1.5	0.9–2.5	3.7	2.8–4.8	4.6	2.3–9.2^c^	3.3	2.6–4.2
Number of residents		p = 0.010		p < 0.001		p = 0.007		p < 0.001
1	0.4	0.1–1.5^c^	1.6	1.0–2.5	2.0	1.1–3.6	1.6	1.1–2.2
2	0.7	1.4–30.5^c^	3.2	2.5–4.2	5.1	3.2–7.9	3.1	2.4–3.9
≥ 3	2.1	1.3–3.2	3.7	3.0–4.5	5.2	3.8–6.9	3.6	3.0–4.2
Situation of dependency by age								
Under 5 years		p = 0.034		p = 0.088		p = 0.194		p = 0.337
Presence	0.8	0.4–1.7	2.8	2.1–3.7	6.2	4.2–9.0	3.2	2.6–4.1
Absence	1.9	1.2–3.0	3.6	3.0–4.3	3.9	2.9–5.3	3.2	2.7–3.8
Under 15 years		p = 0.026		p = 0.008		p = 0.041		p < 0.001
Presence	2.5	1.4–4.3	3.4	2.7–4.2	5.5	4.1–7.4	3.6	3.0–4.4
Absence	1.0	0.6–1.6	3.3	2.7–4.0	3.7	2.6–5.2	2.8	2.4–3.3
65 years or over		p = 0.179		p = 0.429		p = 0.346		p = 0.879
Presence	2.1	1.2–3.5	3.4	2.7–4.2	3.5	2.2–5.4	3.1	2.5–3.8
Absence	1.6	0.9–2.7	3.4	2.8–4.0	4.9	3.8–6.5	3.3	2.8–3.9
Under 15 years, 65 years or over		p < 0.001		p = 0.097		p = 0.294		p = 0.010
Presence	2.4	1.5–3.8	3.3	2.7–4.0	4.8	3.6–6.3	3.4	2.9–4.0
Absence	0.5	0.3–1.0	3.5	2.8–4.4	4.3	2.8–6.5	2.9	2.4–3.6
General	1.7	1.1–2.5	3.4	2.9–3.9	4.6	3.6–5.9	3.2	2.8–3.8

^a^ The percentages shown were weighted by the sample weights.

^b^ Brazil Economic Classification Criterion developed by the Brazilian Research Association (CCEB 2013/ABEP). Available from: http://www.abep.org

^c^ We recommend caution in the interpretation of the data.

Not paying the bills was the main strategy used to deal with the CHE. Saving money was the strategy whose use by different ABEP classes had statistically significant difference (p < 0.05) (Table 4).

**Table 4:** Strategy declared to deal with catastrophic health expenditure. PNAUM, Brazil, 2014.

Strategy used to deal with the expenditure^c^	Strategy declared to deal with catastrophic health expenditure^a,b^								

	Total		A/B		C		D/E		p
			
	%	95%CI	%	95%CI	%	95%CI	%	95%CI	
Not paying the bills	36.0	30.4–42.1	30.1	16.6–48.2	33.1	27.1–39.6	45.8	35.4–56.5	0.082
Taking out a loan from financial institutions	30.0	25.4–35.0	36.7	24.7–50.6	30.8	25.2–37.1	25.0	17.8–33.9	0.274
Taking out a loan from friends or family	19.5	15.5–24.4	11.3	6.8–18.3	20.2	15.1–26.6	21.3	13.7–31.7	0.273
Selling goods	15.4	11.7–20.0	19.9	9.9–36.1^d^	14.1	9.6–20.3	16.5	10.6–24.8	0.626
Not buying food	13.5	10.8–16.7	6.9	3.3–13.7^d^	12.7	9.8–16.4	18.2	12.0–26.5	0.052
Saving money	2.8	1.8–4.2	1.9	0.7–5.1^d^	3.9	2.4–6.2	0.5	0.2–1.6^d^	0.001
Others	0.5	0.2–1.6^d^	1.7	0.4–7.1^d^	0.5	0.2–1.6^d^	0.2	0–1.6^d^	0.053

^a^ The percentages shown were weighted by the sample weights.

^b^ Brazil Economic Classification Criterion developed by the Brazilian Research Association (CCEB 2013/ABEP). Available from: http://www.abep.org

^c^ Not exclusive options, the respondents could indicate as many options as they wanted.

^d^ We recommend caution in the interpretation of the data.

## DISCUSSION

The prevalence of CEM followed the same distribution of CHE as for the regions of the Country, the presence of three or more residents and the presence of a resident in situation of economic dependency. The situations they most reported as being of catastrophic expenditure were the presence of three or more residents and, above all, the presence of a resident in a situation of dependency under 15 years.

Not buying food or paying the bills and saving money were the strategies with greater differences between the economic classes, even though the last one should be interpreted with caution because of the low number of reports. Not paying the bills was the most used strategy by classes C and D/E, while taking out a loan in financial institutions was more common for the class A/B.

This study was conducted with data from national survey, the first one specific for access to and use of medicines with national scope, representative of large regions of the Country, having been included households located in urban areas.

We found convergent values with the average of 3.3 inhabitants per household and 10.8 one-person households reported by the Brazilian Institute of Geography and Statistics (IBGE) in the 2010 census^b^. Unlike the census data, which showed a decline in the number of residents with increased income in households with earnings, we found higher proportion of one-person households in the class D/E. Camarano^10^ signals to the increased proportion of older people living alone, especially women.

The dependency ratio expresses the relationship between the population that has no formal conditions to contribute economically (people under 15 years and those aged 65 years or over) in relation to the population economically active. The situation of economic dependency is usually linked to care dependency, as both children and disabled older people require support to carry out their daily activities^18^. This care will require, among other things, additional economic resources from their guardians to afford the required infrastructure. The catastrophic expenditure, both on health and on medicine, in all the socioeconomic classes, was greater in more numerous families, especially the ones with young residents in a situation of economic dependency.

The CHE level reported was less than that of countries of all income groups (World Bank classification) in a study in which the variable was measured in a similar way^24^ and found moderate means of 44.1%; 29.8%; 18.6%; and 13.4% of households with CHE report, respectively, for countries with low, medium-low, medium-high, and high income. The same study showed that CHE was inversely proportional to the proper functioning of the health services and access provided by governments. Brazil has high rates of access to health services, which makes the proportion of those who needed and did not seek (16.8%) greater than of those who searched and could not be assisted (2.5%)^c^. This indicates that, in general, people can overcome the barriers of access and obtain care, although not necessarily in a timely manner.

The proportions of catastrophic expenditure on health and medicines found converge with other national measures. Study with data from the National Household Budget Survey of 2002/2003^2^, which measured the CHE based on the proportion of consumption and household income, indicated levels smaller than those of international studies. Another national study^12^, using data from two consecutive Household Budget Surveys (HBS) (2002/2003 and 2008), found that painkillers, influenza, and antihypertensive drugs were the therapeutic groups that had the highest participation in the budget of the families who spent money on medicines. The first and third are, at the time of completion of the study, included both in the public supply of medicines in health units and in the Popular Pharmacy for free. It is reasonable to assume that the low level of catastrophic expenditure we found for health and medicines is the result of public policies aiming to expand the access to medicines, despite the many problems that still exist in the pharmaceutical field^15^ ^,^ ^20^. Study conducted in Rio Grande do Sul corroborates the potential protective role of free provision regarding expenditures on medicines, especially in the poorest groups^6^.

In the case of medicines, studies have generally found high rates of access^19^, but while most health care is obtained from the Brazilian Unified Health System (SUS)^22^, most medicines are obtained from the private sector^8^ ^,^ ^17^. The medicines represent a high proportion of expenditure on health and, similarly, in this study they were present in most situations of CHE, with a higher catastrophic expenditure among the poorest, pattern observed in other studies^6^ ^,^ ^7^.

Among the regions, Southeast showed the best situation, both for expenditure on health and on medicines. This finding converges with other studies, which usually indicate better results in this area of the Country, as well as the South region^11^, which, however, did not have the same kind of outcome in our study.

As limitations, we can highlight the option, important to other analyses, of putting the key question for the catastrophic expenditure in the block of household information. However, this resulted, in the case of our approach, in the impossibility of verifying the association with aspects in the individual level, such as reported diseases, health insurance coverage, or use of health services. It could have happened, for example, that in a household with reports of catastrophic expenditure only healthy individuals were interviewed, however, the catastrophic expenditure could have been caused by an individual who was not drawn to answer the questionnaire. The expenses abandoned because of health expenditure, question used as the main filter in the analysis, may have included unnecessary items, judgement however that is permeated by values of difficult consensus. The study was applied only in urban areas, however, this has been prevalent in population concentrations since 1970, reaching 84.4% in 2010^b^. Nevertheless, it cannot be overlooked that, though small, a portion of the Brazilian population lives in rural areas, which, generally, present worse health situation and less use of health services^b^ ^,^ ^d^. The loss of 912 households (4.5%) can be considered small, and unlikely to have affected the findings. Finally, it is important to indicate that we used an indirect measurement of catastrophic expenditure, since we did not ask directly about expenses. On the other hand, there is no formal definition for CEM, so the measurement used in this study considered its impact on the families, whether they needed extra resources or deprived themselves of other goods to obtain care and medicines.

We considered the number of residents dependent on the income, the region of the Country, and the presence of residents in a situation of dependency. For the latter, although we found in the literature two different age limits^e^ ^,^ ^f^ for the definition of old-age dependency, we preferred working with the limit of 65 years for being convergent with the international literature^g^.

Catastrophic expenditures on health and on medicines were present, respectively, in 5.3% and 3.2% of the Brazilian households. Multi-person households, presence of residents in a situation of economic dependency, and belonging to the class D/E had the highest prevalence of CEM. Among the regions, Southeast showed the lowest prevalence. Although the problem is important, with relevant aspects of inequity, the Brazilian policies seem to be protecting families from catastrophic expenses on health and on medicines, because the prevalence found in this study is lower than that found in other countries.

## Footnotes

b Instituto Brasileiro de Geografia e Estatística. Censo demográfico 2010: características da população e dos domicílios: resultados do universo. Rio de Janeiro (RJ): IBGE; 2011.

c Instituto Brasileiro de Geografia e Estatística. Pesquisa nacional por amostra de domicílios. Rio de Janeiro (RJ): IBGE; 2008.

d Instituto Brasileiro de Geografia e Estatística. Pesquisa Nacional de Saúde 2013: percepção do estado de saúde, estilos de vida e doenças crônicas - Brasil, Grandes Regiões e Unidades da Federação. Rio de Janeiro (RJ): IBGE; 2014.

e Rede Interagencial de Informações para a Saúde (RIPSA). Fichas de Qualificação da RIPSA – 2012: Demográficos. Razão de dependência: A.16 – 2012. Brasília (DF): Datasus, 2016 [cited 2016 Aug 1]. Available from: http://157.86.8.70:8080/handle/icict/44737

f Instituto Brasileiro de Geografia e Estatística. Indicadores sociais mínimos: conceito. Rio de Janeiro (RJ): IBGE; 2016 [cited 2016 Aug 10]. Available from: http://www.ibge.gov.br/home/estatistica/populacao/condicaodevida/indicadoresminimos/conceitos.shtm

g The World Bank. Age dependency ratio (% of working-age population). The Washington (DC): World Bank Group; 2016 [cited 2016 Aug 1]. Available from: http://data.worldbank.org/indicator/SP.POP.DPND

## References

[1] Acuña C, Marin N, Mendoza A, Emmerick ICM, Luiza VL, Azeredo TB (2014). Determinantes sociales de la exclusión a los servicios de salud y a medicamentos en tres países de América Central. Rev Panam Salud Publica.

[2] Barros AJ, Bastos JL, Dâmaso AH (2011). Catastrophic spending on health care in Brazil: private health insurance does not seem to be the solution. Cad Saude Publica.

[3] Barros AJD, Bertoldi AD (2008). Out-of-pocket health expenditure in a population covered by the Family Health Program in Brazil. Int J Epidemiol.

[4] Berki SE (1986). A look at catastrophic medical expenses and the poor. Health Aff.

[5] Bermudez J (2014). Acesso a medicamentos: direito ou utopia?.

[6] Bertoldi AD, Barros AJD, Camargo AL, Hallal PC, Vandoros S, Wagner A (2011). Household Expenditures for Medicines and the Role of Free Medicines in the Brazilian Public Health System. Am J Public Health.

[7] Boing AC, Bertoldi AD, Barros AJD, Posenato LG, Peres KG (2014). Desigualdade socioeconômica nos gastos catastróficos em saúde no Brasil. Rev Saude Publica.

[8] Boing AC, Bertoldi AD, Boing AF, Bastos JL, Peres Karen Glazer (2013). Acesso a medicamentos no setor público: análise de usuários do Sistema Único de Saúde no Brasil. Cad Saude Publica.

[9] Boing AC, Bertoldi AD, Posenato LG, Peres KG (2014). The influence of health expenditures on household impoverishment in Brazil. Rev Saude Publica.

[10] Camarano AA (2004). Os novos idosos brasileiros: muito além dos 60?.

[11] Duarte EC, Schneider MC, Paes-Sousa R, Ramalho WM, Sardinha LMV, Silva-Júnior JB, Castillo-Salgado C (2002). Epidemiologia das desigualdades em saúde no Brasil: um estudo exploratório.

[12] Garcia LP, Sant’Anna AC, Magalhães LCG, Freitas LRS, Aurea AP (2013). Gastos das famílias brasileiras com medicamentos segundo a renda familiar: análise da Pesquisa de Orçamentos Familiares de 2002-2003 e de 2008-2009. Cad Saude Publica.

[13] Knaul FM, Wong R, Arreola-Ornelas H, Méndez O (2011). Household catastrophic health expenditures: a comparative analysis of twelve Latin American and Caribbean countries. Salud Publica Mexico.

[14] Leive A (2008). Coping with out-of-pocket health payments: empirical evidence from 15 African countries. Bull World Health Organ.

[15] Mendes LV, Campos MR, Chaves GC, Silva RM, Freitas PS, Costa KS (2014). Disponibilidade de medicamentos nas unidades básicas de saúde e fatores relacionados: uma abordagem transversal. Saude Debate.

[16] Mengue SS, Bertoldi AD, Boing AC, Tavares NUL, Silva Dal Pizzol T da, Oliveira MA (2016). Pesquisa Nacional sobre Acesso, Utilização e Promoção do Uso Racional de Medicamentos (PNAUM): métodos do inquérito domiciliar. Rev Saude Publica.

[17] Mengue SS, Tavares NUL, Costa KS, Malta DC, Silva JB da (2015). Fontes de obtenção de medicamentos para tratamento de hipertensão arterial no Brasil: análise da Pesquisa Nacional de Saúde, 2013. Rev Bras Epidemiol.

[18] Muszyńska MM, Rau R (2012). The old-age healthy dependency ratio in Europe. J Popul Ageing.

[19] Paniz VMV, Fassa AG, Facchini LA, Bertoldi AD, Piccini RX, Tomasi E (2008). Acesso a medicamentos de uso contínuo em adultos e idosos nas regiões Sul e Nordeste do Brasil. Cad Saude Publica.

[20] Paniz VMV, Fassa AG, Facchini LA, Piccini RX, Tomasi E, Thumé E (2010). Free access to hypertension and diabetes medicines among the elderly: a reality yet to be constructed. Cad Saude Publica.

[21] Simonsen M, Skipper L, Skipper N (2009). Price sensitivity of demand of prescription drugs: exploiting a kinked reimbursement scheme.

[22] Viacava F, Souza PRB, Szwarcwald CL (2005). Coverage of the Brazilian population 18 years and older by private health plans: an analysis of data from the World Health Survey. Cad Saude Publica.

[23] Vialle-Valentin CE, Serumaga B, Wagner AK, Ross-Degnan D (2015). Evidence on access to medicines for chronic diseases from household surveys in five low- and middle-income countries. Health Policy Plan.

[24] Wagner AK, Graves AJ, Reiss SK, LeCates R, Zhang F, Ross-Degnan D (2011). Access to care and medicines, burden of health care expenditures, and risk protection: results from the World Health Survey. Health Policy.

[25] Wyszewianski L (1986). Financially catastrophic and high-cost cases: definitions, distinctions, and their implications for policy formulation. Inquiry.

[26] Xu K, Evans DB, Carrin G, Aguilar-Rivera AM, Musgrove P, Evans T (2007). Protecting households from catastrophic health spending. Health Aff.

[27] Xu K, Evans DB, Kawabata K, Zeramdini R, Klavus J, Murray CJ (2003). Household catastrophic health expenditure: a multicountry analysis. Lancet.

